# The Performance Changes and Migration Behavior of PLA/Nano-TiO_2_ Composite Film by High-Pressure Treatment in Ethanol Solution

**DOI:** 10.3390/polym12020471

**Published:** 2020-02-18

**Authors:** Zhenya Tang, Fangling Fan, Chunli Fan, Kai Jiang, Yuyue Qin

**Affiliations:** 1 Faculty of Environmental Science and Engineering, Kunming University of Science and Technology, Kunming 650550, China; zytang@kust.edu.cn; 2 School of Energy and Environment Science, Yunnan Normal University, Kunming 650550, China; ffl333@126.com; 3 Institute of Agriculture and Food Engineering, Kunming University of Science and Technology, Kunming 650550, China; fanchunli1995@163.com (C.F.); cuirui910@163.com (K.J.)

**Keywords:** poly (lactic acid), nano-TiO_2_, high pressure treatment, migration

## Abstract

To study the relationship between performance changes and nanoparticles migration of the composite film at different migration stages, the poly (lactic acid) (PLA)/nano-TiO_2_ composite film treated by high pressure was immersed in 50% (*v*/*v*) ethanol solution for 45 days at 40 °C, and the film characteristics and migration behavior were analyzed. The results showed that the migration of the composite film with the highest loading of nano-TiO_2_ (20 wt. %) in alcoholic food simulated solution was far less than 10 mg/kg during the 45-day migration process. Although with the increase of migration time, the micro-morphology of composite film became rougher, the crystallinity decreased and the gas permeability increased, but the internal crystal structure of the composite film remained basically unchanged. The PLA/nano-TiO_2_ composite films treated by high pressure treatment were relatively stable, and had good performance and migration behavior in alcoholic food simulated solution, the nanocomposite film after high pressure treatment could be used to reduce nano-TiO_2_ particle migration and subsequently reduce human exposure as the packaging film for the packaging of alcoholic food, which provide a theoretical basis for the applications of high pressure treatment of PLA/nano-TiO_2_ composite films in food packaging material and broaden its application prospects.

## 1. Introduction

In recent years, countries around the world have been responding to the concept of sustainable development, promoting harmony between man and nature, and maintaining ecological balance, which have become an important trend of the world’s green development. Among them, solving the pollution of packaging such as plastic has become the top priority in protecting the environment. As a result, scientists and researchers are keen to develop new biodegradable materials [1,2,3]. Poly (lactic acid) (PLA) is a kind of polyester made from starch, cellulose, and other carbohydrates by hydrolysis, fermentation, purification, and polymerization [4,5]. It could be biodegraded to produce CO_2_ and H_2_O, realizing natural circulation. It is an ideal green macromolecule material. In addition, PLA has excellent thermal processability and biocompatibility, but PLA also has its own shortcomings, such as: high brittleness, no antibacterial property, etc. [6]. Therefore, without changing the original properties of PLA, researchers have carried out a lot of modification studies to explore and find PLA-based composites that can meet different application requirements.

In recent years, with the continuous improvement of people′s living standards and the upgrading of consumer market, food safety issues are increasingly becoming the focus of attention, so under this circumstance, nano-packaging materials have gradually come into people′s vision. By adding nanoparticles into packaging materials, nano-packaging materials are modified and compounded, thus giving new materials antibacterial properties, mechanical properties and air permeability [7,8,9]. Nano-TiO_2_ is the most important one among many nano-materials. Because of its inert, non-toxic, and photocatalytic antibacterial properties, nano-TiO_2_ has been widely used in packaging materials for food, with good antibacterial and fresh-keeping effects [10,11,12]. At the same time, it has been approved by the Food and Drug Administration (FDA) for use in the food packaging [13]. For example, Lian et al. found that nano-TiO_2_ was added to PVA–CHI (Polyvinyl alcohol–chitosan) films, not only the water vapor and gas barriers were improved, but also the mechanical properties and antimicrobial properties were effectively enhanced [14]. Zhang et al. prepared chitosan-nano-TiO_2_ composite films. The results showed that the addition of nano-TiO_2_ could not only improve the hydrophilicity of the composite films, but also make the composite films have good antimicrobial activity [15].

Although the properties of materials and the shelf life of food are improved by adding nanoparticles, with the further research on the biological safety of nanoparticles, it has been proved that nanoparticles in food packaging materials tend to migrate to food during the packaging process, which may cause potential safety hazards [16,17,18]. Moreover, there are few reports about the changes of the properties of food packaging materials after nanoparticles migration at home and abroad.

High pressure processing technology is a new type of non-thermal food processing technology. Its application in food industry mainly includes food sterilization, improving texture, and prolonging shelf life of stored food [19,20,21]. At present, there are few reports on high pressure processing of food packaging materials, so high pressure technology has a good application prospect in the field of food packaging materials. The relationship between the performance change of the composite film at different migration stages and the amount of migration of nanoparticles by high pressure treatment needs further study.

In previous studies, we found that the strength and permeability of PLA/nano-TiO_2_ composite films were effectively improved after 300 MPa high pressure treatment [22,23,24,25]. However, the performance change of composite film during migration, the migration amount of nano-TiO_2_ under high pressure and the exposure risk have not been studied. In this work, PLA/nano-TiO_2_ composite films with different mass fractions after high pressure treatment were immersed in alcoholic food simulated solution (ethanol 50%, *v*/*v*) for 45 days at 40°C. The purpose of this study was to compare different TiO_2_ mass fractions (0, 1, 5, 10, 15, and 20 wt. %) of PLA nanocomposite films after high pressure treatment on the properties of nanocomposite films during contacting with alcoholic food simulated solutions. The scanning electron microscopy (SEM), X-ray diffraction (XRD), differential scanning calorimetry (DSC), water vapor permeability (WVP), and oxygen transmission rate (OTR) were measured, and the migration test was also studied.

## 2. Materials and Methods 

### 2.1. Materials 

Poly (L-lactic acid) (PLA, *M*_w_ = 280 kDa, *M*_w_/*M*_n_ = 1.98) was purchased from Shenzhen Jinyu Technology Co., Ltd. (Shenzhen, China). The nano-TiO_2_ (Purity of 99.5% and average particle size < 100 nm) was obtained from Nanjing Muke Nanotechnology Co., Ltd (Nanjing, China). The reagents used in this experiment, such as dichloromethane and concentrated nitric acid, were purchased from Tianjin Biochem Pharmaceutical Co., Ltd (Tianjin, China).

### 2.2. Preparation and High Pressure Treatment of PLA-Based Composite Films

The PLA-based nanocomposites containing different proportions of nanoparticles were made by solvent evaporation. Firstly, 2 g PLA and nano-TiO_2_ powder with different mass fractions (0, 1, 5, 10, 15, and 20 wt. %) were accurately weighed and dissolved in 50 mL dichloromethane, and then placed on a magnetic stirrer for overnight at room temperature. Subsequently, the conical bottle was placed in the ultrasonic wave for 30 min, in order to make the nanoparticles disperse uniformly in the PLA mixture as far as possible. Finally, the homogeneous solution was poured onto the polytetrafluoroethylene board (20 cm × 20 cm). After overnight, after volatilization of all dichloromethane, the composite film can be obtained by slowly peeling off the board. The composite film was placed in a vacuum bag, vacuumed and treated for 10 min under 300 MPa with a high pressure treatment apparatus. The composite films were put into (23 ± 2) °C and (50 ± 5)% relative constant temperature and humidity dryers, waiting for use. Among them, the composite films with 0, 1, 5, 10, 15 and 20 wt. % nano-TiO_2_ content were named as PLA, PLA/Ti1, PLA/Ti5, PLA/Ti10, PLA/Ti15, and PLA/Ti20 films, respectively.

### 2.3. Migration Test

According to EU regulations and FDA standards, 50% (*v*/*v*) ethanol solution is selected as a food simulated solution to represent alcoholic foods [25]. The migration of nano-TiO_2_ to food simulated solution at 40 °C after high pressure treatment of PLA/ nano-TiO_2_ composite film was studied. Each composite film was cut into squares (4 cm × 4 cm) size with scissors and accurately weighed. The sample was put into a bottle with 30 ml of 50% (*v*/*v*) ethanol solution. All of the bottles containing the samples and ethanol solution were sealed and transferred into a constant temperature and humidity chamber at 40°C for 45 days. In this study, 0, 5, 10, 15, 25, 30, and 45 days were selected as the migration time, and the content of TiO_2_ in 50% (*v*/*v*) ethanol solution was determined by ICP-OES (Optima 4300DV, Perkin Elmer, Shelton, CT, USA) [14]. Calibration solution was prepared by dilution of stock standard solution. For each sample, the migration experiment was carried out in triplicate and the results are expressed in mg/kg.

### 2.4. Measurement of Distribution Coefficient and Diffusion Coefficient

Most of the migration models of compounds in macromolecule materials are developed on the basis of the Fick diffusion law. According to the actual situation of nano-TiO_2_ migration, the Fick’s second law is used to establish the migration mathematical model of the PLA/nano-TiO_2_ composite film by high pressure treatment. Among them, diffusion coefficient (*D_P_*) and distribution coefficient (*K*) are two very important parameters in the process of establishing the mathematical model of migration. Diffusion coefficient refers to the rate at which substances diffuse from composite films to food simulated solutions, which is related to the physical and chemical properties of the components inside the composite film, external factors and the nature of food contact. Its expression is shown in Formula 1:

(1) $${\left[\frac{1}{\pi }-\frac{1}{\alpha }\frac{M_{F,t}}{M_{P,0}}\right]}^{0.5}=-\frac{D_{P}^{0.5}}{\alpha }\frac{t^{0.5}}{L_{P}}+\frac{1}{\pi^{0.5}}$$

In the formula, *M_F,t_* is the amount of nano-TiO_2_ migrated to food simulated solutions at t time (mg), *M_P,0_* is the initial content of nano-TiO_2_ (mg), and *L_P_* is the thickness of composite film (cm).

Distribution coefficient refers to the ratio of the concentration of migrant substances in food simulated solutions to that in composite films when the migration amount reaches equilibrium. Its expression is shown in Formula 2:

(2) $$K_{P/F}=\frac{C_{P,e}}{C_{F,e}}=\frac{M_{P,0}-M_{F,e}}{C_{F,e}V_{P}}\;$$

In the formula, *C_P,e_* and *C_F,e_* is the equilibrium concentration of nano-TiO_2_ in the composite film and food simulated solutions respectively (mg/cm^3^), *M_F,e_* is the equilibrium content of nano-TiO_2_ in food simulated solutions (mg), and *V_P_* is the volume of the composite film (cm).

### 2.5. Conditional Assumptions

According to previous studies, the establishment of migration mathematical models requires the following assumptions:

(1) Before the migration experiment, nano-TiO_2_ is evenly distributed in the composite film, and none of nano-TiO_2_ is found in the food simulated solutions.

(2) nano-TiO_2_ enters the food simulated solutions from both sides of the composite film.

(3) The food simulated solutions is stirred evenly, and the concentration of nano-TiO_2_ in the food simulated solutions was the same everywhere.

(4) In the process of migration, *D_P_* and *K* are constants.

(5) The migration and diffusion of nano-TiO_2_ in composite films conform to the Fick’s law.

(6) At the interface between the composite film and the food simulated solutions, the migration is balanced at any time.

(7) There is no interaction between the composite film and the food simulated solutions, and the boundary layer effect of the composite film is neglected.

### 2.6. Migration Model

The migration process of the PLA/nano-TiO_2_ composite films by high pressure treatment is mainly based on Fick’s second diffusion law model, in which the Piringer equation is a relatively mainstream migration diffusion model:

(3) $$\frac{M_{F,t}}{M_{F,\infty }}=1-\overset{\infty }{\underset{n=0}{\sum }}\frac{8}{{\left(2n+1\right)}^{2}\pi^{2}}exp\left[-\frac{{\left(2n+1\right)}^{2}\pi^{2}}{4L_{p}^{2}}Dt\right]\;\;\;$$

Among them, *M_F,t_* is the amount of nano-TiO_2_ migrated to food simulated solutions at t time (mg), $M_{F,\infty }\;$is the amount of nano-TiO_2_ migrated to food simulated solutions at equilibrium (mg), *L_p_* is the thickness of composite film (cm), and *D* is the diffusion coefficient of nano-TiO_2_ in composite film (cm^2^/s).

### 2.7. Scanning Electron Microscopy (SEM)

The composite film was fractured by placing tweezers in liquid nitrogen. The brittle fracture surfaces of the composite films were observed by SEM (NOVA, Chengdu, China). In a high vacuum environment, the test operating voltage was 10 kV to observe the brittle fracture profile.

### 2.8. X-Ray Diffraction (XRD)

The structure of nanocomposite films was characterized by Brucker D8 Advance X-ray diffraction. Among them, the X-ray source in this experiment is Cu Kα radiation. The range of the diffraction angle 2*θ* is 10°–60°, the testing time is about 7 min, the working voltage is 40 kV, and the working current is 40 mA.

### 2.9. Differential Scanning Calorimetry (DSC)

Using 214 Polyma differential scanning calorimeter for analysis, various parameters for testing the thermal properties of the film were obtained (Thermal Analysis of German Netzsch, Selb, Germany). 10 mg of film was introduced in an aluminum pan. During the heating process, the temperature rises from 10 to 200 °C, and the heating rate is 10 °C/min, which lasts for 5 min at 200 °C. This is to eliminate the residual solvents and some uncertainty factors. Then the temperature falls to 10 °C at the rate of 20 °C/min. For the second run, the temperature rises to 200 °C at the rate of 10 °C/min. The curve of the second run was recorded to obtain the glass transition temperature (*T_g_*), cold crystallization temperature (*T_c_*), melting temperature (*T_m_*) and crystallinity degree (*X_c_*). Formula 4 is used for calculating *X_c_* [23]:

(4) $$X_{c}\;=\;\frac{\Delta H_{m}\;-\;\Delta H_{c}}{\Delta H_{m}^{0}\times w}\times 100$$

Among them, $\Delta H_{m}$ is the melting enthalpy (J/g), $\Delta H_{c}$ is the cold crystallization enthalpy (J/g),$\;\Delta H_{m}^{0}$ is the melting enthalpy (93.7 J/g) of pure PLA when it is fully crystallized, and w is the mass fraction of PLA in the composite film.

### 2.10. Water Vapor Permeability (WVP)

The WVP value of the films was obtained from ASTME96-95 standard method and corrected appropriately [24]. First, accurately weigh 10 grams of anhydrous silica gel and place it in the weighing bottle (40 mm × 25 mm), fix the film sample to the weighing bottle, and then place the weighing bottle in the desiccator with saturated NaCl at the bottom. Finally, the dryer is placed in a constant temperature and humidity environment (25 °C/65% relative humidity). The weighing bottle was measured once every 1 hour, and observed for 12 hours, and the weight change of the weighing bottle was calculated. The formula for calculating WVP is:

(5) $$\mathrm{WVP}\;=\;\frac{W\times X}{A\times \;T\;\times \Delta P}$$

In the formula, *W* is the weight loss of every sample test (g), *X* is the thickness of the composite film (m), *A* is the effective area of the composite film enclosing the weighing bottle (m^2^), *T* is the time interval (s), and ΔP is the vapor pressure difference (Pa) on both sides of the sample. Each film was tested for three times.

### 2.11. Oxygen Transmission Rate (OTR)

The OTR value of the composite films was measured using an oxygen analyzer (Oxy Sense 5250i, Zillion Co., Ltd, Shanghai, China). The film was bonded to the sample chamber with a sealant, and passed nitrogen into the upper half of the sample chamber for 5 min to make sure that the air is completely removed, then passed pure oxygen to the lower part of the sample chamber for testing. Each film was tested three times and averaged.

## 3. Results and Discussion

### 3.1. Migration Amount of Nano-TiO_2_

The migration amount of nano-TiO_2_ depends on the relationship between the content of nano-TiO_2_ in the 50% (*v*/*v*) ethanol solution and the amount of nano-TiO_2_ embedded in the composite film [25]. A 50% (*v*/*v*) ethanol solution was chosen as the alcoholic food simulated solution to detect the migration of nano-TiO_2_ in food simulated solution.

**Figure 1:**
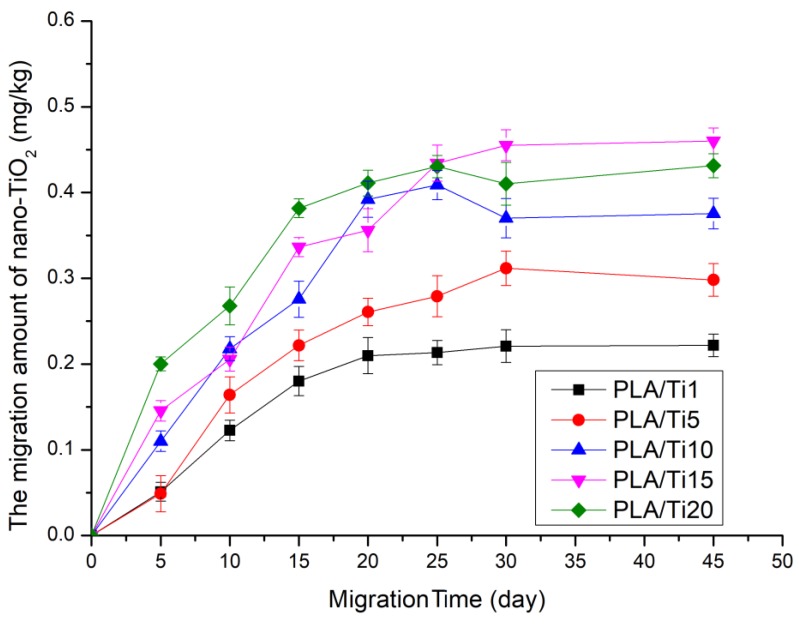
The amount of nano-TiO_2_ migrated to food simulated solution after high pressure treatment.

During the migration process, the content of nano-TiO_2_ migrated into the food simulation solution was shown in Figure 1. The thickness of PLA, PLA/Ti1, PLA/Ti5, PLA/Ti10, PLA/Ti15, and PLA/Ti20 film was 45, 46, 48, 50, 51, and 52 μm, respectively. It could be found from Figure 1 that the migration amount of nano-TiO_2_ to food simulated solution increased with the increase of time, and the migration amount was proportional to the migration time. In the early stage of migration experiment, the migration of nanoparticles showed a rising state and reached a stable state in the 20th–45th days. This is due to the fact that nano-TiO_2_ on the surface of the composite film has not completely entered the PLA matrix, which was easier to migrate to the simulated solution in the early stage of migration, and the nanoparticles exposed on the brittle fracture surface of the composite film first migrate to the simulated solution, and then the nanoparticles embedded in the PLA would migrate under the action of the simulated solution, which was consistent with our previous research results [26].

In Figure 1, it was also found that on day 45, the migration of nano-TiO_2_ particles into food simulated solutions in PLA/Ti20 films treated by high pressure was (0.43 ± 0.01) mg/kg, which was far lower than the regulation given by the European Food Safety Authority (Maximum migration of Ti particles in food packaging materials is 10mg/kg). In previous studies, we found that the maximum migration content of PLA/Ti20 films that were not treated with high pressure was (0.54 ± 0.04) mg/kg on day 45 [27], which indicated that that high pressure treatment affected the migration of nanoparticles in the composite film and reduced the migration performance of the films. The reason for this phenomenon was that the crystallinity of the composite film was increased after high pressure treatment. As pressure increases, the segment movement of the amorphous zone was enhanced. Then, the chain motion was weakened after the pressure was released. The molecular chains were tightly packed to form a crystalline structure of the polymer packaging material. When the composite film was treated under high pressure, the crystallinity of the composite film increased [28].

### 3.2. Mathematical Model of Migration

The diffusion coefficient (D_P_) and distribution coefficient (K) of PLA/nano-TiO_2_ composite film by high pressure treatment were shown in Table 1. It could be found that the D_P_ value increased with the increase of nano-TiO_2_ content, while the K value decreased with the increase of nano-TiO_2_ content. This indicated that the migration of compounds in PLA followed the rule that the smaller the distribution coefficient was, the larger the diffusion coefficient would be. At the same time, the higher the content of nanoparticles in the composite films was, the easier the nanoparticles migrated from composite film to the food simulated solutions. This further proved that the higher the content of nanoparticles was, the greater the migration of compounds would be. These results indicated that the chemical content of the material was the main factor affecting the migration behavior in the same food simulated solutions.

Since the composite film in this experiment is a PLA monolayer composite film, the volume of the composite film with migration behavior is much smaller than that of the food simulated solutions. According to Fick diffusion theory, Formula 3 can be simplified to Formula 6.

(6) $$\frac{M_{F,t}}{M_{F,e}}=1-\frac{8}{\pi^{2}}\exp\left(\frac{-\pi^{2}Dt}{L_{P}^{2}}\right)$$

The D_P_ obtained in Table 1 was introduced into the Formula 6 to obtain the migration–diffusion equations with different nano-TiO_2_ content. The predicted trend of the migration model was deviated from the true value, and the predicted trend value of the migration model was larger than the experimental value (Table 2). This was due to the expansion of PLA/nano-TiO_2_ composite films in varying degrees during the long immersion process, and the boundary effect between packaging film and food simulated solutions during migration process will also have an impact on the results.

### 3.3. Microstructure

**Figure 2:**
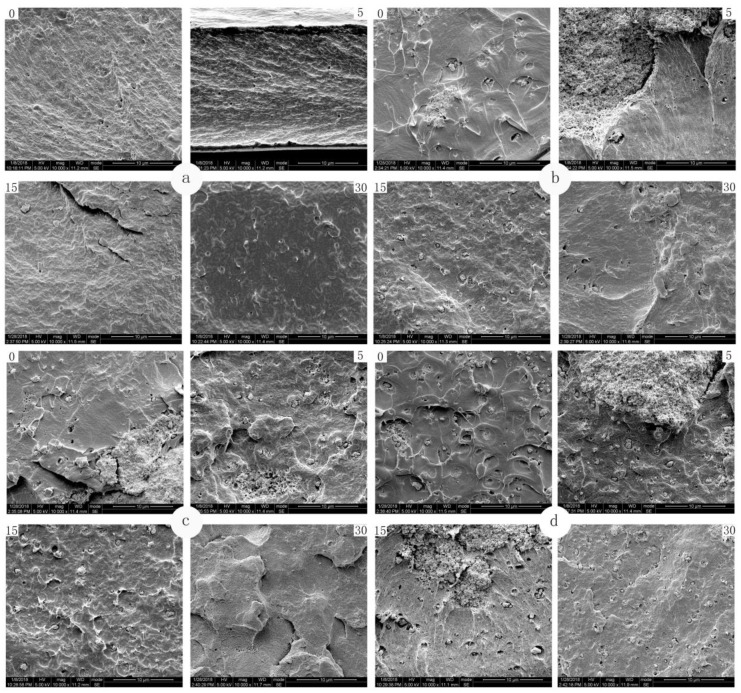
Scanning electron microscopy (SEM) images of (**a**) PLA, (**b**) PLA/Ti5, (**c**) PLA/Ti10, and (**d**) PLA/Ti20 composite films treated by high pressure contacting with ethanol solution on day 0, 5, 15, and 30 (magnification: 10000×).

As shown in Figure 2, the microstructures of four high-pressure treated composite films during immersion were studied. In Figure 2a, the morphology of the micro-section of pure PLA film after different immersion time showed that the high-pressure treatment made many depressions on the section of composite film, which made the cross-section became very pleated, and this trend became more and more obvious with the increase of composite film soaking time. On the 30th day, it could be seen from the picture that micro-pore also appeared on the pure PLA composite film. It can be found from Figure 2b–d that the nanocomposite films after high pressure treatment were more susceptible to the influence of food simulated solution. After 15 and 30 days of migration and immersion, obvious pleats, holes and even collapses gradually appeared in the section of the micro-section of the composite film.

In previous studies, it was found that before immersion, the micro-section of PLA basement film showed a uniform, continuous and compact structure [22]. After being soaked in the food simulated solution, the migration of the nanoparticles from the composite film system and the migration of the solvent molecules of the food simulated solution into the composite film system simultaneously occurred, and the ethanol solution entered the composite film system due to the low polarity of the ethanol solution. Moreover, due to the low polarity of the ethanol solution, it entered into the composite film system and interacted with the PLA molecules, so that the interaction between the macromolecules was strengthened and the chains were contracted and gathered. With the increase of soaking time, the food simulated solution continued to move into the composite film, which resulted in the rearrangement and aggregation of PLA macromolecular chains in the film. There was a phase separation interface between the chain aggregates and other areas of the film. Therefore, holes or depressions were formed during the fracture process.

### 3.4. Crystalline Structural Changes

**Figure 3:**
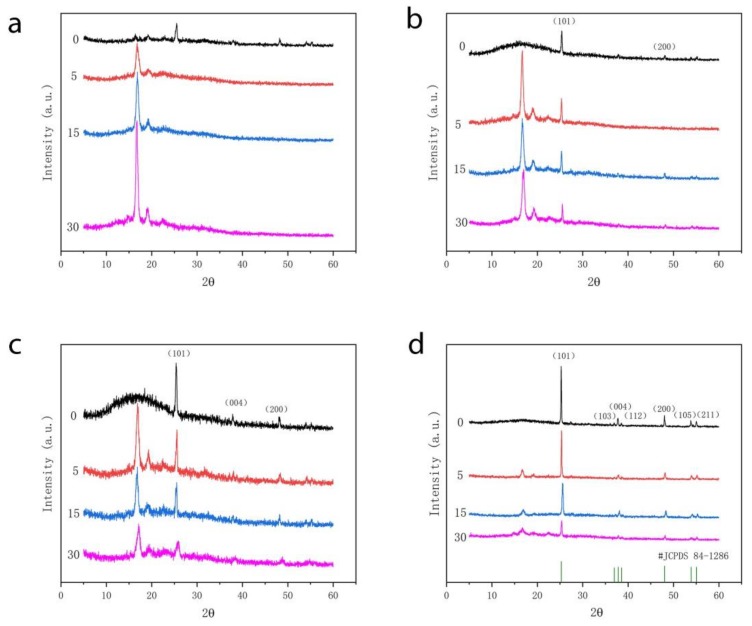
X-ray diffraction (XRD) curves of (**a**) PLA, (**b**) PLA/Ti5, (**c**) PLA/Ti10, and (**d**) PLA/Ti20 composite films treated by high pressure contacting with ethanol solution on day 0, 5, 15, and 30.

After being soaked in the alcoholic food simulated solution, the XRD pattern of the PLA-based film material treated by high pressure was shown in Figure 3. When the diffraction angle of the composite film was 2θ = 16.8°, the diffraction peaks were wider, which indicated that the PLA matrix was in amorphous structure. It could be seen from Figure 3 a–d that when the films were immersed in the simulated solution on the 5th day, two smaller diffraction peaks appeared at the diffraction angles of 2θ = 15.5° and 19.1°. They were the phase 200/100 and phase 210 planes of PLA-specific orthorhombic crystalline form, respectively [29]. It could also be seen from the figure that there were diffraction peaks near 2θ = 25.36°, 2θ = 37.77°, 2θ = 47.99°, and 2θ = 54.21°, which conformed to the standard spectrum of TiO_2_ (JCPDS #84-1286) [16].

From Figure 3, it could be seen that the XRD peak position of the composite film did not change significantly with the soaking time in the food simulated solution, but the characteristic peak (2θ = 25.36°) of nano-TiO_2_ decreased (Figure 3b–d). For PLA/Ti20 films, the intensity of the characteristic peak of nano-TiO_2_ was reduced by half on the 30th day, but the intensity of nano-TiO_2_ was still the highest in the 30th day. This was because that the interaction force between nanoparticles and PLA macromolecules was weakened after high pressure treatment. It was easier to migrate to the food simulated solution system. The content of nanoparticles in the composite film decreased, so the peak intensity at the characteristic peak of TiO_2_ decreased. This was consistent with previous migration results. In addition, as the migration time increased, the peak value increased at 2θ = 16.8°. The characteristic crystalline form of PLA becomes obvious as the simulated solution gradually moves into the composite film system and nanoparticles move into the simulated solution system during the migration process.

### 3.5. Thermal Performance

**Figure 4:**
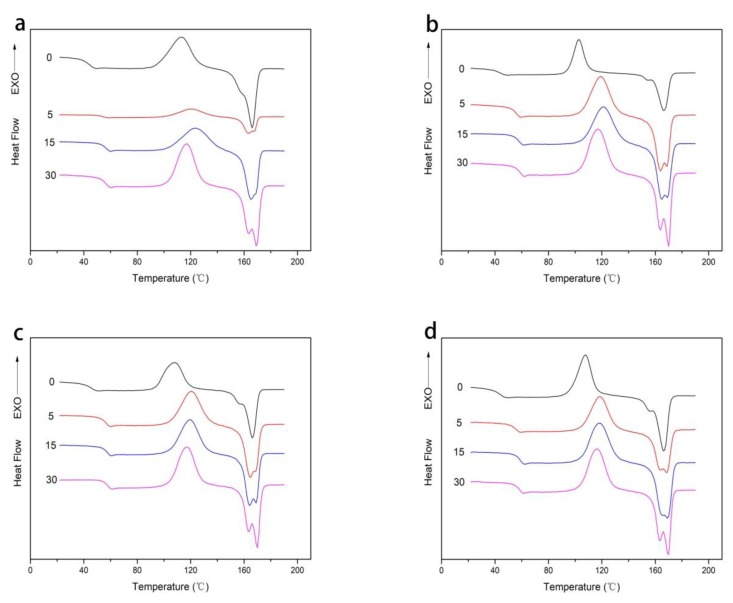
Differential scanning calorimetry (DSC) curves of (**a**) PLA, (**b**) PLA/Ti5, (**c**) PLA/Ti10, and (**d**) PLA/Ti20 composite films treated by high pressure contacting with ethanol solution on day 0, 5, 15, and 30.

The DSC curves of four composite films after high pressure treatment at different migration time were shown in Figure 4. It could be found that the thermodynamic properties (*T_g_*, *T_c_*, and *T_m_*) of the composite films treated by high pressure did not change significantly with the increase of nanoparticles. However, the crystallinity of PLA firstly increased and then decreased from PLA to PLA/Ti20, which indicated that the addition of nano-TiO_2_ could increase the crystallinity of PLA, but excessive nanoparticles might lead to their aggregation in polymer matrix, resulting in the decrease of its crystallinity. Doganay et al. found that the thermodynamic properties of the new PLA-based nanocomposites formed by mixing nano-silver particles with PLA did not change significantly [30]. Cortes et al. also found that the thermodynamic properties of the new composite materials obtained by mixing nano-silver particles with polyethylene materials by heating and melting did not change significantly compared with ordinary pure polyethylene materials [31].

The thermal performance parameters of the PLA/nano-TiO_2_ composite films treated with high pressure at different migration time were shown in Table 3. It was found from Figure 4a–d that the migration of composite films in food simulated solution would affect the thermodynamic properties of the composite films. Except that there was no significant change in *T*_m_, the other thermodynamic parameters were improved. In terms of *T*_g_, the *T*_g_ of the composite film increased significantly from 46.0, 47.6, 46.4, 48.8 to 59.0, 66.6, 60.2, and 61.7 °C, and the *T*_c_ increased from 119.2, 105.4, 106.6, 108.6 to 128, 127.2, 124.2, and 127.7 °C, respectively. The nucleating ability of nano-TiO_2_ and the hydrolytic chain cleavage preferentially proceeded in the amorphous region [10,11,12]. However, with the prolongation of soaking time, *T*_g_ and *T*_c_ firstly increased and then decreased. This is because in the initial process of migration, nanoparticles interact with the surrounding overall force (van der Waals force), to simulate the solution to migrate into composite film, and with the increase of time, the composite film in the process of hydrolytic degradation of polymer chain rupture can lead to the formation of different crystal structure. PLA film emerged distinct double melting peaks on day 30. However, PLA/Ti5, PLA/Ti10, and PLA/Ti20 composite film emerged double melting peaks from day 5. The presence of double melting peaks in PLA composites is attributed to the melting crystalline phase formed during the second heating run [32]. It could also be seen from Table 3 that from day 0 to day 30, the crystallinity of the film in contact with the food simulated solution gradually decreased. This was due to the increase of fluidity of polymer chains, which was conducive to the diffusion of ethanol to the amorphous phase with irregular structure, and destroyed the internal structure of the composite film, thus leading to the change of crystallinity of PLA [33].

### 3.6. Barrier Performances

The WVP and OTR are very important indicators of food packaging materials. A certain barrier performance can reduce the gas transmission between food and the external environment, to ensure food quality and extend the shelf life. The WVP and OTR of the four films after high pressure treatment at four migration stages were shown in Table 4. It could be found from the table that at the same migration time, the values of WVP and OTR decreased at first, and then increased with the increase of nanoparticle content. The addition of a certain amount (5 wt. %) of nano-TiO_2_ particles into PLA matrix would increase the path of gas entering and leaving the composite film material, resulting in the decrease of WVP and OTR [18]. However, when the content of nanoparticles continued to increase, nano-TiO_2_ particles would make the surface rougher than PLA/Ti5 film. This was conducive to the flow of gas into and out of the packaging material, leading to the subsequent increase of WVP and OTR.

It could also be seen from Table 4 that the barrier properties of nanocomposite films with the same mass fraction decreased with the increase of migration time. This was because the internal structure of PLA-based film was uniform and continuous before immersion, and after immersion in food simulated solution, nanoparticles migrate from the composite film and ethanol molecules migrated into the composite film at the same time. When nanoparticles migrate out, the interaction force between nanoparticles and PLA molecules weakens, resulting in the decrease of crystallinity and loosening of internal structure of the composite film, which ultimately leads to the decrease of gas barrier performance and makes the gas easier to enter and exit between the internal and external environment of the composite film.

## 4. Conclusions

To investigate the effect of high pressure treatment on the performance changes and potential migration of nano-TiO_2_ particle from PLA/nano-TiO_2_ food composite film, the ethanol solution was used to simulate the real environment of the composite film material packaging food and multiple techniques were used. In the migration analysis, it was found that the migration of nano-TiO_2_ in alcoholic food simulated solution increased dramatically in the first few days and remained stable for 20–45 days, and its maximum migration amount was much lower than that stipulated by the European Food Safety Authority. Although with the increase of immersion time, the micro-morphology of the composite film became more and more rough, the crystallinity of the composite film decreased and the permeability of the composite film increased, but the crystal structure of the composite film did not change.

This study confirmed that the nanocomposite film after high pressure treatment could be used to reduce nano-TiO_2_ particle migration and subsequently reduce human exposure as the film for the packaging of alcoholic food and no food safety risk in the food processing process. This provided a theoretical basis for the applications of high pressure treatment on PLA/nano-TiO_2_ composite films as food packaging materials.

## Figures and Tables

**Table 1:** The distribution coefficient and diffusion coefficient of Poly (lactic acid) (PLA)/nano-TiO_2_ composite films by high pressure treatment.

Sample	K	D_P_ (10^−16^cm^2^/s)
PLA/Ti1	5.36	2.43
PLA/Ti5	4.14	3.34
PLA/Ti10	2.64	4.17
PLA/Ti15	2.09	4.32
PLA/Ti20	1.36	4.85

**Table 2:** Migration model equation of PLA/ nano-TiO_2_ composite film by high pressure treatment in food simulated solution.

Sample	Migration Model Equation
PLA/Ti1	M_F,t_ = 0.241 − 0.196 exp[−(9.59*10^−9^)t]
PLA/Ti5	M_F,t_ = 0.334 − 0.272 exp[−(1.32*10^−8^)t]
PLA/Ti10	M_F,t_ = 0.417 − 0.339 exp[−(1.64*10^−8^)t]
PLA/Ti15	M_F,t_ = 0.441 − 0.359 exp[−(1.71*10^−8^)t]
PLA/Ti20	M_F,t_ = 0.508 − 0.413 exp[−(1.91*10^−8^)t]

**Table 3:** The thermal properties of PLA/nano-TiO_2_ composite films after high pressure treatment.

Sample	Time	*T*_g_ (°C)	*T*_c_ (°C)	*T*_m_ (°C)	*X*_c_ (%)
PLA	Day 0	46.0	119.2	171.3	20.5
	Day 5	59.0	128.0	170.1	18.3
	Day 15	58.8	128.3	171.3	16.5
	Day 30	59.9	121.5	169.4	15.2
PLA/Ti5	Day 0	47.6	105.4	170.0	23.8
	Day 5	66.6	127.2	170.5	21.2
	Day 15	67.0	125.5	169.0	18.3
	Day 30	65.5	123.0	171.2	16.8
PLA/Ti10	Day 0	46.4	106.6	171.4	26.7
	Day 5	60.2	124.2	170.2	24.5
	Day 15	61.5	115.0	171.5	22.9
	Day 30	60.8	119.3	170.9	21.2
PLA/Ti20	Day 0	48.8	108.6	170.0	25.4
	Day 5	61.7	127.7	169.4	23.1
	Day 15	62.9	121.0	170.0	21.4
	Day 30	62.5	119.0	170.5	19.4

**Table 4:** Water vapor permeability (WVP) and oxygen transmission rate (OTR) of PLA/nano-TiO_2_ composite films after high pressure treatment.

		Time (Day)			
	Sample	0	5	15	30
WVP	PLA	4.81 ± 0.17^a^	5.13 ± 0.11^a^	5.34 ± 0.22^a^	5.55 ± 0.15^a^
(g·m)/(m^2^·s·Pa)	PLA/Ti5	3.46 ± 0.32^b^	4.26 ± 0.22^b^	4.58 ± 0.14^b^	4.81 ± 0.25^b^
	PLA/Ti10	4.12 ± 0.15^ab^	4.83 ± 0.18^ab^	5.05 ± 0.18^ab^	5.49 ± 0.09^a^
	PLA/Ti20	4.97 ± 0.18^a^	5.24 ± 0.09^a^	5.45 ± 0.09^a^	5.61 ± 0.13^a^
OTR	PLA	4.02 ± 0.18^a^	4.35 ± 0.14^a^	4.54 ± 0.21^a^	4.77 ± 0.15^ab^
[(cm^3^/(24h*m^2^)]*(cm/bar)	PLA/Ti5	2.92 ± 0.13^b^	3.32 ± 0.12^b^	3.61 ± 0.11^b^	3.99 ± 0.13^c^
	PLA/Ti10	3.28 ± 0.09^b^	3.58 ± 0.17^b^	3.84 ± 0.17^b^	4.21 ± 0.12^bc^
	PLA/Ti20	3.98 ± 0.21^a^	4.24 ± 0.12^a^	4.52 ± 0.15^a^	4.81 ± 0.20^a^

^a–b^ Values followed by different superscripts in the same column denote significant difference (*p* < 0.05), where a is the highest value.
